# Application of 3D–printed and patient-specific cast for the treatment of distal radius fractures: initial experience

**DOI:** 10.1186/s41205-017-0019-y

**Published:** 2017-11-09

**Authors:** Yan-Jun Chen, Hui Lin, Xiaodong Zhang, Wenhua Huang, Lin Shi, Defeng Wang

**Affiliations:** 1 Institute of Clinical Anatomy, School of Basic Medical Sciences, Southern Medical University, Guangzhou, China; 2 Research Center for Medical Image Computing, Department of Imaging and Interventional Radiology, The Chinese University of Hong Kong, Prince of Wales Hospital, Shatin, NT Hong Kong; 3 Department of Medicine and Therapeutics, The Chinese University of Hong Kong, Shatin, NT Hong Kong; 4 Chow Yuk Ho Center of Innovative Technology for Medicine, The Chinese University of Hong Kong, Shatin, NT Hong Kong; 5 Shenzhen Research Institute, The Chinese University of Hong Kong, Shenzhen, China; 6 Department Medical Radiology, The Third Affiliated Hospital of Southern Medical University, Guangzhou, China

**Keywords:** 3D printing, Patient-specific, Orthopaedic cast, Clinical trial, Assessments

## Abstract

**Background:**

Distal radius fracture is common in the general population. Fracture management includes a plaster cast, splint and synthetic material cast to immobilise the injured arm. Casting complications are common in those conventional casting technologies. 3D printing technology is a rapidly increasing application in rehabilitation. However, there is no clinical study investigating the application of a 3D–printed orthopaedic cast for the treatment of bone fractures. We have developed a patient-specific casting technology fabricated by 3D printing. This pioneering study aims to use 3D–printed casts we developed for the treatment of distal radius fractures, to provide the foundation for conducting additional clinical trials, and to perform clinical assessments.

**Method:**

Ten patients with ages between 5 and 78 years are involved in the clinical trial. Patients are applied 3D–printed casts we developed. Orthopaedic surgeons carried out a six-week follow-up to examine clinical outcomes. Two questionnaires were developed for the assessment of clinical efficacy and patients’ satisfaction. These questionnaires are completed by physicians and participating patients.

**Results:**

A 3D–printed cast creates a custom-fitted design to maintain the fractured bone alignment. No loss of reduction is found in all patients. Compartment syndrome and pressure sores are not present. Patient comfort gets positive scores on the questionnaire. All (100%) of the patients opt for the 3D–printed cast instead of the conventional plaster cast.

**Discussion:**

A patient-specific, 3D–printed cast offers a proper fit to immobilise an injured arm and holds the fracture reduction appropriately. A custom-fitted structure reduces the risk of pressure-related complications due to the high and concentrated local stress. The ventilated and lightweight design minimises interference with a patient’s daily activities and reduces the risk of cutaneous complications. Patients express a strong preference for using a 3D–printed cast instead of a plaster cast. Limitations of the novel cast include a slight odour after heavy sweating and the relatively high cost due to the limitations of current 3D printing technologies.

**Conclusions:**

This pioneering study is the first clinical trial on the application of a 3D–printed cast for the treatment of forearm fractures. The novel casting technology heals the fracture effectively without casting complications. The 3D–printed cast is patient-specific and ventilated as well as lightweight, and it features both increased patient comfort and satisfaction.

## Background

Distal radius fractures are common skeletal injuries and occurred at all ages of the general population [1, 2]. These types of fractures are reported as having one of the highest incidences accounting for over 15% of bone fractures [3, 4]. Distal radius fracture management usually includes a plaster cast, splint, and a moulded synthetic material cast to immobilise the injured upper extremity [5–9]. A normal course of the treatment includes the application of a cast and several follow-up clinical visits lasting four to six weeks [7, 10, 11]. The traditional casts are described as having both poor ventilation and an improper fit, while also causing discomfort. These morbidities associated with conventional casts may result in cast complications such as cutaneous diseases, bone and joint injuries, or malunion [7, 12, 13]. The rate of cast-related complications published is high with up to 31% being reported in published studies [14].

The 3D printing technology is rapidly advancing in medical applications [15]. In the manufacture of rehabilitation tools, 3D printing technology is being applied to orthopaedic cast fabrication to create patient-specific features with an appropriate fit and a ventilated structure [16]. Jake Evill and Deniz Karasahin proposed a novel design of casts with a web-like structure and fabricated it by using a 3D printing technique. The cast models were built from 3D–scanned images of subjects’ limbs and created by using computer-aided design software, which can generate a Stereolithography (STL) file, a standard file format widely used for 3D printing. Kim et al. developed a hybrid model of wrist orthosis composed of a 3D–printed frame and an injection moulding shell [16, 17]. Although 3D printing has made advances at a rapid pace in the development of casting techniques, all published technologies for the design and fabrication of 3D–printed casts are still in the concept stage without clinical application [16]. To date, there is no clinical study investigating the application of 3D–printed casts [18].

Our previous published study has developed a rapid and intelligent technique to create a patient-specific orthopaedic cast fabricated by 3D printing [16]. Orthopaedic surgeons with little knowledge of engineering can design a high-quality cast in a short amount of time by utilising the technique. The novel cast also has additional features such as being ventilated and hygienic advantages that potentially lower the risk of complications. To our knowledge, there is no published clinical trial using the 3D–printed cast for the treatment of distal radius fractures. This study is to perform a pioneer clinical trial for the casting technology utilising 3D–printed casts for the treatment of distal radius fractures.

## Methods

Ten patients (age range from 5 to 78 years old) including four males and six females are involved in the clinical trial. There were six patients who suffered distal radius and ulnar styloid fractures. Distal radius fractures were present in three patients. One patient sustained fractures of both the distal radius and ulna. Exclusion criteria included pathological/open fractures, fractures requiring internal fixation and patients who were not available for local follow-up. All clinical trials were performed in southern China where the weather was humid and warm, normally above 30 degrees Celsius during the clinical testing period. Patients first underwent closed reduction using traditional plaster cast fixation due to tissue swelling. The first treatment stage lasted for one week after swelling subsided. 3D–printed orthopaedic casts developed by our published techniques [16] were applied to these patients after one week fixation. Two follow-up examinations and investigations were performed about the second and sixth week after the application of the 3D–printed cast [11, 19].

**Fig. 1:**
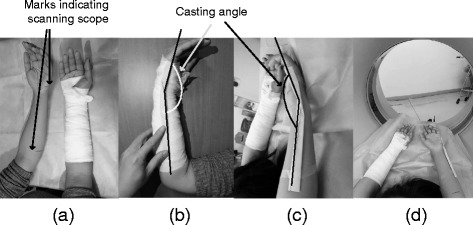
Scanning method: (**a**) determine the scanning scope by making marks on the patient’s arm; (**b**) bend the wrist at the casting angle; (**c**) the patient lies on the table and bends both wrists at the casting angle; (**d**) scans are taken of both hands

Patients’ limb injury data are obtained from computerised tomography (CT) scanning or magnetic resonance imaging. An injured patient is first examined using radiography to identify the fracture type and locate the region of the injury. Physicians then perform the closed reduction followed by plaster casting in the initial phase. To obtain workable data for later cast design, both arms are scanned by a CT imaging system (Aquillion CX 64, Toshiba, Japan) or MR (Achieva 1.5 T, Philips) imaging equipment. The 3D scanning system using a 3D scanner, which was employed in our previous study [16], is not utilised in the clinical trial due to the difficulty of the scanning procedure. To use a 3D scanner, patients must keep their arms at a position without movement for several minutes. This procedure is difficult to conduct. In China, the use of CT or MR imaging is recommended for further diagnosis and treatment of the injury. Before scanning, a technician makes marks on the patient’s arm to indicate the scanning scope as shown in Fig. 1(a). The technician helps the patient to bend his wrist at an angle (Fig. 1 (b)), which is termed a casting angle, where the casting arm keeps the wrist at the same angle during the treatment period. The value of the casting angle is determined by an orthopaedic surgeon according to the injury details. The patient lies on the patient table of the scanning system, and then raises and bends his wrist at the casting angle (Fig. 1(c)). Both hands with symmetric postures are scanned to obtain raw data (Fig. 1(d)). Data from the other forearm without injury can be the alternative due to the swelling of the injured forearm during the initial inflammation stage.

To evaluate the clinical trial, we designed questionnaires concerning clinical efficacy and patient satisfaction [6, 7, 20]. Orthopaedic surgeons and patients worked together to complete the questionnaire after seven weeks of treatment. The design of these questionnaires is based on the existing questionnaire used in the hospital performing the clinical test and published studies [7]. Two questionnaires have been designed including two groups of survey questions associated with treatment-related issues and patient satisfaction assessments [7]. The first group lists four survey questions related to clinical effect based on expert opinions and previous investigation carried by Chinese orthopaedic surgeons (Table 1). Pressure sore is an important assessment item that is a common complication caused by traditional casting [14]. Other common complications, such as the stability of immobilisation, the severity of compromised blood flow, and pressure-related patient discomfort, are also included in the assessment. The first questionnaire is completed by the surgeon who exams patients at the second and sixth week after the application of a cast. Patient satisfaction or patient sensation and behaviour is another assessment including comfort, patient compliance, preference between 3D–printed and plaster cast, cast odour, and skin itchiness (Table 2). As a pilot study, the assessment of the patients’ preference addresses patients’ opinions about the physical structure and wearer comfort. The inflammation-induced uncomfortable experience, especially during the initial stage of treatment, should be ruled out from the assessment. With the assistance from doctors, patients complete the second assessment questionnaire after the second and seventh weeks of casting. Doctors only explain the details of the questionnaire to let the patient fully understand questions without any personal recommendations to affect selection.

**Table 1:** Assessment of clinical effectiveness of a 3D–printed cast

Assessment Item	Assessment contents and grading standard			
	excellent-3	good-2	acceptable-1	poor-0
Stability of immobilisation	No loss of reduction	Slight shift but no need for re-manipulation	Reinforced same cast	Loss of reduction requiring further procedure
Blood circulation	Good terminal circulation with a florid complexion	Venous obstruction relief after physical movement or arm lifting	Pale skin, low temperature of the arm	Significant ischaemia of involved limb, compartment syndrome
Wear-pressure-related pain	No pain	Slight pain with a minor influence on sleep	Mild pain caused poor-quality sleep	Severe pain caused difficulty falling asleep
Pressure sores	No abnormality of the skin	Non-blanchable erythema of the intact skin	Skin breakdown or bleeding blister	Full thickness skin loss

**Table 2:** Patient satisfaction questionnaire

Item	Assessment contents and grading standard			
	Excellent-3	Good-2	Acceptable-1	Poor-0
Patient comfort	Very comfortable	Occasional irritation	Medium irritation	Bad experience wearing cast
Patient compliance	Strong willingness to try new cast	Minor doubt	Dubious but complied	Accepted reluctantly
Patient preference between 3D–printed cast and conventional plaster cast	Opted for 3D–printed cast without hesitation	Preferred a 3D–printed cast to a plaster cast	Either cast is acceptable	Insisted on using conventional cast
Cast odour and smell	None	Slight cast odour	Smelly cast after heavy sweating	Stinky cast
Skin itchiness	No itch	Rarely itchy	Frequent itch but tolerable	Severely itchy

**Fig. 2:**
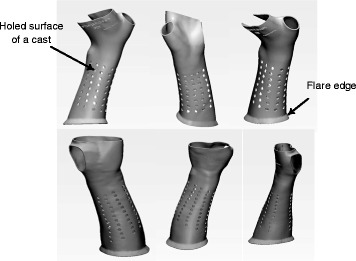
The cast model designed by our developed techniques

*Patients’ raw models are input into the intelligent system developed by our previously published study to perform patient-specific design as per clinical requirements* [16]*. Short arm casts are designed for the treatment of distal radius fractures. A cast model is designed as a holed surface pattern with a flare on the lower edge near the elbow for the consideration of ventilation and wearer-friendly features (*Fig. 2 *).*

**Fig. 3:**
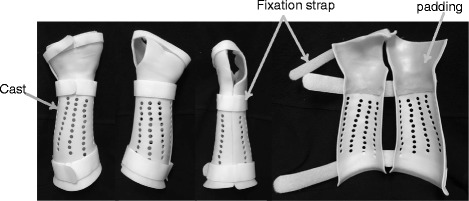
Post-processing includes adding the fixation strap and padding on the specific anatomical regions close to the wrist and ulnar head

Medically compatible materials such as polypropylene (PP) and polyamide (PA2200) are employed in the 3D printing fabrication of an orthopaedic cast. These materials are China Food and Drug Administration (CFDA) approved as Class I materials for rehabilitation devices. We utilise selective laser sintering (SLS) 3D printer EOS P395 (Germany) or Stereolithography (SLA) printer RS4500 (UnionTech, China) to produce the cast. Technically, post-processing for a 3D–printed cast is necessary for producing a final physical model. It includes padding a 3D–printed cast, mechanical grinding or rolling sharp edges, and adding fixation components. As shown in Fig. 3, fixation straps are mounted on the cast to adjust the assembly and create a cast that is tailor-fitted to an injured limb. In particular, cushion pads are glued on some specific anatomical regions, such as the wrist and ulnar styloid process, to avoid local high pressure and scratching of the skin.

## Results

**Fig. 4:**
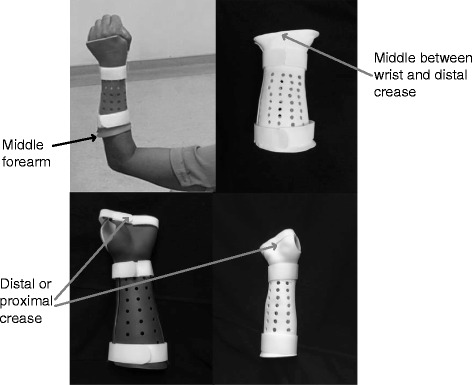
A short arm cast extends from the mid-forearm to the distal or proximal crease. To consider the mobilisation of the wrist for a slight injury, a short arm cast can also extend from the mid-forearm to just above the wrist crease

A personalised and 3D–printed cast is fabricated as a split structure with two half parts but still keeps the circumferential structure when applied to an injured extremity. A short arm cast extends from the mid-forearm to the distal, proximal crease [8] (Fig. 4). In some mild cases with slight injuries of a forearm, a short arm cast can extend from the mid-forearm to the middle area between the wrist and distal crease (Fig. 4).

**Fig. 5:**
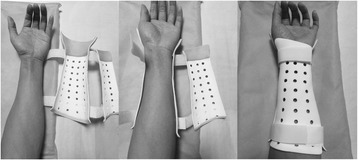
The application procedure of a 3D–printed cast with a split structure. The split design and Velcro strap allow the cast to be adjusted and have custom-fitted features

All patients prefer the custom-fit feature that establishes comfortable contact on the injured arm. The surrounding Velcro straps allow the adjustment of the assembly of the cast to accommodate the swelling forearm in the initial inflammatory phase of a fracture (Fig. 5). The 3D–printed cast satisfies the orthopaedic requirement for the treatment of a fracture in terms of the seven-week follow-up. The novel cast maintains fracture bone alignment and immobilises the forearm during the healing process. No loss of reduction is found in all participating patients. Moreover, no breakage occurred in any cast during the treatment period.

**Fig. 6:**
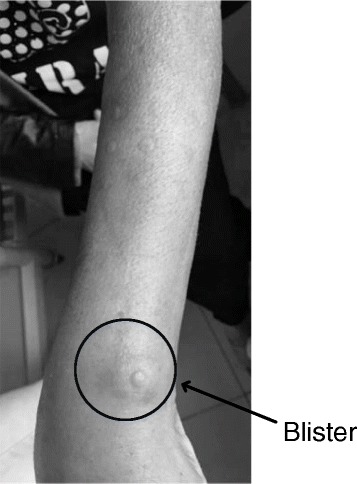
A blister caused by high local pressure near the region of the ulnar head

Pressure sores, common casting complications occurring in a traditional cast, were not present in any patients. However, one female patient (age 56, height 5′1″, weight 41 kg) had a blister with around 5 mm diameter on the bony prominence near the head of the ulna (Fig. 6). The patient adjusted the cast by herself without consulting with a physician. It then gave rise to a tight fit. No pain and no complaints were reported from the patient. The blister has disappeared one day after a physician adjusted the cast. A skinny arm can be easily bruised from the cast with relatively tight contact. This complication, skin breakdown, was not present in any patients even though thin patients were involved in the clinical trial.

Patients were taught how to check for compartment syndrome by themselves within one week of the application of the 3D–printed cast. As one of the most serious complications of traditional casting techniques, compartment syndrome was not found in any of the patients examined in the second and sixth week after casting. Orthopaedic surgeons inspected the skin appearance under the cast, and no visual signs of pressure damage were found in these patients. Complications associated with compromised blood flow commonly occurring when using circumferential casts and splints were not present during the healing period.

The first group of assessment (Table 1), clinical efficacy, was scored as 9.8 out of 12 and 30% of patients scored this group lower than 9 of the score. The lowest score was 8 out of 12 in the first group evaluation (Table 3). The second group of the assessment (Table 2), patient satisfaction to the application of the novel cast, was scored as 11.5 out of 15 (Table 3). In this questionnaire, 80% percent of patients scored the new casting technique over 11 out of 15, and no patients scored fewer than 8. Both assessments of patient comfort and comparison between a plaster and a 3D–printed cast were assigned full marks. However, patient compliance was assigned as 2.1 out of 3 where the lowest score was rated.

**Table 3:** Assessment results

	Clinical efficacy	Patient satisfaction
Mean score	9.8/12	11.5/15
Highest score	11/12	14/15
Lowest score	8/12	9/15

## Discussion

An appropriate casting technique not only holds the fracture reduction at a proper anatomic position but also minimises the risks of complications related to distal fracture. Complications including cutaneous diseases, compartment syndrome and vascular comprise, have been reported in conventional cast application due to the unbalanced pressures and high stiffness [3, 12]. Traditional casting creating mould utilising plaster and thermoplastic has rigid structure without flexibility and poor ventilation. In addition, the swelling of soft tissue occurring in an injured forearm at the initial stage makes it difficult to create patient-specific features. 3D–printed casts are featured as patient-specific and fully ventilated as well as lightweight structures [16]. 3D–printing technology is an image-based technology combined with rapid prototyping that can create a patient-specific cast in terms of injury regions and severity. An orthopaedic surgeon performs the closed reduction for a displaced fracture followed by casting fixation. Patient-specific features of casting play an important role to maintain the alignment and avoid loss of closed reduction. Moreover, the custom-fit structure ensures the matching surface geometry between the cast and arm and thus disperses pressure. The ventilated structure featured in the novel cast confers the benefits of improved patient comfort and reduced risk of cutaneous complications.

Acquisition of a patient’s image is an important process for performing cast design. In the imaging process, fracture patients are required to hold a special posture to keep the forearm or lower leg in a natural position. Participants with fractures have swollen arms where the original shapes are difficult to be discerned. This study proposes the mirror technique, which scans the counterpart of an injured limb. Eight out of 10 testing casts used the contralateral arm. Bilateral symmetry exists in natural biological structures. For example, surgeons assume the bilateral symmetry of the human skeletal system for surgery purposes. Also, Islam et al. demonstrated the symmetric morphology of some human bones [21]. Thus, the mirror technique offers relatively accurate patient data and minimises imaging difficulties for the patient.

**Fig. 7:**
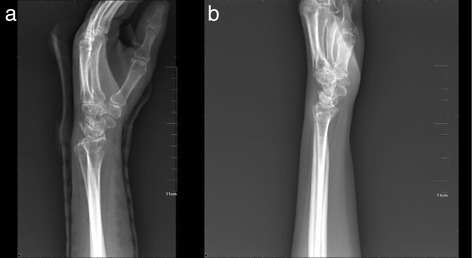
**a** A fractured forearm fitted with a cast; (**b**) the recovery of the broken arm with a superior clinical outcome

All patients participating in these clinical trials have completed the entire therapeutic course without negative clinical consequences. Rather, the patients in these trials had superior clinical outcomes (Fig. 7). No clinical trial using 3D–printed cast has been reported. The 3D–printed cast offers custom-fitted immobilisation during the entire treatment process. A cast with a correct fit can apply appropriate orthopaedic pressure on the injured arm to maintain bone alignment even after significant deformities occurred in the soft tissue. No patients found the loss of reduction in these clinical trials due to the technique that ensured they were fitted correctly.

Pressure-related complications are reported with traditional casts, especially circular casts. The cast utilised for this study are designed as splitting and circular structures with padding components, which can split the cast to accommodate the injured limb and relieve pressure. Slightly slow circulation is found after two weeks of cast application due to the wearing pressure. Participated patients can relieve the symptom through raising the injured arms. Slight pain is developed from wearing pressure, but no complaint is reported from patients. Wearing pressures are necessary for any casting or splint technology to maintain the reduction of the fracture and perform orthopaedic corrections effectively [22]. Patient-specific and 3D–printed casts develop proper fit by the shape of the injured arm and thus reduce the risk of stress concentration since large contact areas are created. Concentrated stress is considered as an important factor associated with compartment syndrome. These technical advantages are present in the clinical trial so that no cases have compartment syndrome.

**Fig. 8:**
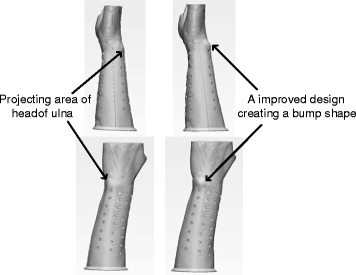
Projecting areas like the head of ulna or radius bone have an increased risk of developing high local wearing pressure under a cast. An improved design creates a bump shape to avoid direct contact between the cast and the underlying skin

Both clinicians and patients are highly concerned about pressure sores developing beneath the cast. To a patient with a distal radius or Colles’ fracture, pressure sores frequently develop in some regions of a forearm, such as on the heads of the radius or ulna, due to their protruding shapes. High local stresses generated from wearing pressure occur in such regions. In the early two weeks of cast application, pink but not broken skin arises on the regions of heads of the radius or ulna accounting for 20% of participating patients using a 3D–printed cast without a padding layer. In addition, even though custom-fit features are created, a slight motion between the injured arm surface and the cast is common regardless of the type of the cast application. The pink skin on those regions results from high local pressure and motion-related scratch. Two approaches to improve design have been proposed in the 3D–printed cast: creating bump shape on those regions and padding the regions as shown in Fig. 8. Eight out of 10 patients were applied those improved casts. Moreover, the material of cast with high stiffness potentially developed a discomfort contact to the skin. Those two improvements potentially reduce the risk of high local pressure and gain clinical benefits to solve pressure-related skin problems. The improvements bring sound clinical outcomes that no reddened or pink skin arises from the casting arm.

**Fig. 9:**
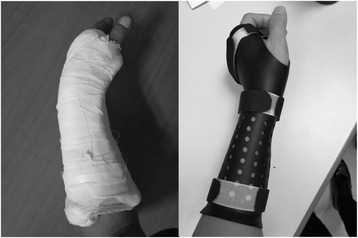
The cumbersome structure of a conventional plaster cast compared with a 3D–printed cast. Patients express a strong preference to utilise a 3D printed cast instead of a conventional cast

As a novel cast patients and clinicians never used, a 3D–printed cast gets high credit from patients with its ventilated, comfortable and fashionable design. All patients assign the highest score on the assessment of comfort. The patient-specific structure creates moderate contact like a fitted sleeve covering the injured arm and prevents injuries from external impacts and scratches. The ventilated structure keeps the microenvironment dry between the skin and the cast and reduces the risk of cutaneous complications. It should be noted that the lightweight structure still maintains a high level of strength. Engineering analysis performed in our previous study demonstrated that the structure of the printed cast was able to resist either normally imposed loads or accidentally impact loads [16]. In this study, breakage due to poor strength of the structure was not found in any of the cases. The wearer-friendly design minimises the interference with daily activities of the patient. Patient compliance or treatment adherence is a challenge for a physician to carry out this research. The details of treatment technologies and potential side effects were informed by the physician. In the early stage of the clinical trial, most patients who show curiosity rather than suspicion on the application of the 3D–printed cast were mostly attracted by the novel 3D printing technology with non-interventional treatment. A few patients were sceptical since no such technology had been used in clinical applications. After two weeks of application of the novel cast, 100% of patients opted to utilise a 3D–printed cast instead of a plaster cast (Fig. 9).

There are some limitations of this study concerning casting technology and clinical testing. First, acquisition of a patient’s data is a challenge for a patient with a fracture. A patient is asked to keep a required position during scanning for the purpose of data integrity. Additionally, we use mirror technology to acquire the counterpart of the fractured arm. Variation between arms is shown in the normal anatomy. Further investigations should be required to evaluate the bilateral symmetry of human bones digitally. There is no complaint about the fitting issue by using the mirror technique. The availability of biocompatible materials for 3D printing fabrication is limited. We used polypropylene (PP) and polyamide (PA2200) with Young’s modulus 1300Mpa. As our experiences, a material with stiffness higher than 2000Mpa of Young’s modulus, like acrylonitrile butadiene styrene (ABS), may cause uncomfortable contact to the skin. In addition, although a ventilated structure has been created, physicians receive a few of complaints about the smelly cast from participating patients who sweat a lot in hot weather. It should be noted that all clinical trials were carried out in a southern Chinese city with hot and humid weather during the testing period. The fabrication cost of a 3D printing cast is relatively high, about $150 US Dollars, compared with conventional alternatives less than $50 US Dollars. The rapid growth of 3D printing technologies would reduce the fabrication cost in the near future. As a pilot study, we are mainly concerned with the clinical feasibility of the application of the 3D–printed cast. Due to the limited amount of patients, the initial study investigated a small-sized group without performing statistical analysis for comparison among conventional and 3D–printed casts.

As a new casting technology, we conducted the clinical trials with the focus on mild distal fracture. Cast design and 3D–printing fabrication would require a longer time. In emergency situations requiring a cast for the case of an acute fracture, as per our initial experiences, the 3D–printed cast would not be suggested currently. Its personalised structure and ventilated as well as lightweight design bring about quality patient experiences as confirmed by positive assessments of patient comfort and acceptance. Those two assessments would be helpful for the improvement of the current casting technology. This pilot study provides an initial experience on how to apply the 3D printing technique in the treatment of fractures. The future study would perform clinical trials on a larger scale sample size, and as a comparison study among conventional and 3D–printed casts to assess whether clinical differences were statistically significant.

## Conclusions

This study performs a pilot study with the focus on the clinical trial for the treatment of distal radius fracture using patient-specific and 3D printing cast developed by our published techniques. The novel cast performs circumferential support for the fractured forearm and prevents the injury from external impact. The patient-specific design maintains the alignment of fracture bones and creates the custom-fit and moderate wearing pressure to avoid compartment syndrome and pressure sores, which is considered as the casting complication to challenge conventional casting technology. The ventilated structure and fashionable design of the novel cast combined with the 3D printing fabrication increase patient comfort and satisfaction. Superior clinical outcomes have been obtained from clinical trials.
